# Prenatal exposure to preeclampsia is associated with accelerated height gain in early childhood

**DOI:** 10.1371/journal.pone.0192514

**Published:** 2018-02-13

**Authors:** Johanna Gunnarsdottir, Sven Cnattingius, Maria Lundgren, Katarina Selling, Ulf Högberg, Anna-Karin Wikström

**Affiliations:** 1 Department of Women’s and Children’s Health, Uppsala University, Uppsala, Sweden; 2 Department of Medicine, Clinical Epidemiology Unit, Karolinska Institutet, Stockholm, Sweden; Centre Hospitalier Universitaire Vaudois, FRANCE

## Abstract

**Background:**

Preeclampsia is associated with low birth weight, both because of increased risks of preterm and of small-for-gestational-age (SGA) births. Low birth weight is associated with accelerated childhood height gain and cardiovascular diseases later in life. The aim was to investigate if prenatal exposure to preeclampsia is associated with accelerated childhood height gain, also after adjustments for SGA-status and gestational age at birth.

**Methods:**

In a cohort of children prenatally exposed to preeclampsia (n = 865) or unexposed (n = 22,898) we estimated height gain between birth and five years of age. The mean difference in height gain between exposed and unexposed children was calculated and adjustments were done with linear regression models.

**Results:**

Children exposed to preeclampsia were on average born shorter than unexposed. Exposed children grew on average two cm more than unexposed from birth to five years of age. After adjustments for maternal characteristics including socioeconomic factors, height, body mass index (BMI) and diabetes, as well as for parents smoking habits, infant’s breastfeeding and childhood obesity, the difference was 1.6 cm (95% CI 1.3–1.9 cm). Further adjustment for SGA birth only slightly attenuated this estimate, but adjustment for gestational age at birth decreased the estimate to 0.5 cm (95% CI 0.1–0.7 cm).

**Conclusion:**

Prenatal exposure to preeclampsia is associated with accelerated height gain in early childhood. The association seemed independent on SGA-status, but partly related to shorter gestational age at birth.

## Introduction

Cardiovascular disease is a leading cause of death and imposes a substantial burden on the health care system.[1] According to the Developmental Origins of Health and Disease concept, early life environment induces changes in the development of both a fetus and a child, which may impact future risks of diseases.[2] More than 20 years ago an association between low birth weight and cardiovascular disease was first reported.[3] Recently, the question was raised if also prenatal exposure to preeclampsia is associated with future adverse cardiovascular health in the offspring.[4] Prenatal exposure to preeclampsia is associated with increased risk of hypertension in young adults, [5] also in offspring born with normal birth weight.[4]

Accelerated height gain in children is associated with hypertension in adulthood,[6, 7] and in infants born short, accelerated height gain during childhood is also associated with cardiovascular disease.[8] Accelerated growth in infancy is often seen in children born with low birth weight.[9, 10] Preeclampsia is strongly associated with low birth weight, both because of preterm birth and birth of small-for-gestational-age (SGA) infants.[11, 12] However, it is uncertain if exposure to preeclampsia is associated with accelerated growth in offspring born with normal birth weight.

We hypothesized that exposure to preeclampsia is associated with accelerated height gain during early childhood, also after adjustments for SGA-status and gestational age at birth. We investigated the association between exposure to preeclampsia and growth in height during the first five years of life in a population-based cohort of more than 23,000 children.

## Methods

### Data sources

Uppsala County mother and child database was created by linkage between the Swedish Medical Birth Register, the Uppsala County Child Health Register, the Register of Total Population, and the Register of Education. Individual record linkage was enabled through the personal identity number, uniquely assigned to each Swedish resident at birth or at immigration.

The Swedish Medical Birth Register includes information collected at antenatal visits, during and after delivery until discharge from the hospital. At the first antenatal visit the mother is interviewed about her reproductive history and smoking habits. The mother’s weight is measured and recorded, while maternal height is generally self-reported. After delivery, the mother’s age, the infant’s anthropometric measurements and complications during pregnancy and delivery (according to the International Classification of Diseases (ICD) codes) are recorded.

The Uppsala County Child Health Register includes information collected from visits to child health care units, starting at 1 week of age and ending at six years. Attendance to child health care in Uppsala County is high, where 97% of children have at least six registered visits.[13] Parents are interviewed about breastfeeding that is registered as exclusive, partial and no breastfeeding. The child’s height and weight are measured at 18 months, 3, 4, and 5 years. The height is registered as whole centimeters whereas weight is registered in grams. Anthropometric measurements are not recorded in the Register if the appointments are more than 2 months earlier or later than the planned according to age of the child. Length of newborns and 18 month children (measured in a supine position) will be referred to as height throughout the article.

From the Registers of Total Population and Education, held by Statistics Sweden, we retrieved information on mother’s country of birth and years of formal education, respectively.

### Study population

The study population was children born in Uppsala County 2000–2007 with registered height at five years of age. During this period 31,951 children were born in Uppsala County. We excluded 445 infants exposed to gestational hypertension (ICD-10 code O13), since we suspect that there may be some misclassification between preeclampsia and gestational hypertension. The final population included children with a registered height at 5 years, a total of 23,763 children. Electronic registration of height at child health care started in 2005, and children born before 2003 therefore have incomplete series of height measurements. (Table 1)

**Table 1:** Number of children with height registered at different age by year of birth.

Year of birth	2000	2001	2002	2003	2004	2005	2006	2007
18 months	*251	*315	*389	*1447	2795	2722	2950	2873
3 years	*263	*355	2479	2615	2835	2818	3005	2892
4 years	*322	2323	2561	2710	2924	2829	3005	2958
^§^ **5 years**	**2544**	**2652**	**2844**	**2986**	**3161**	**3106**	**3313**	**3153**

* The cursive numbers represents measurements from before 2005, the year of electronic recording to the Child Health Register.

^§^Bold numbers represents our study population in the analysis of height gain.

### Exposure

The exposure variable was preeclampsia that was defined through the ICD-10 diagnostic codes O14-O15. Severe preeclampsia was defined by O14.1 (severe preeclampsia), O14.2 (HELLP-syndrome) or O15 (eclampsia) whereas mild preeclampsia was defined by O14.0 and O14.9. According to Swedish guidelines the clinical definition of preeclampsia during the study period was a rise in blood pressure (≥140/90 mm Hg measured on at least two subsequent occasions) combined with significant proteinuria (≥ 0.3 g/24 hours or +1 on at least two subsequent occasions or +2 on dipstick). The quality of the diagnosis of preeclampsia in the Birth Register has been validated previously: of 148 pregnancies coded as preeclampsia according to ICD-9 standards in the Birth Register, 137 (93%) had the disease according to the individual records.[14] Number of children exposed to preeclampsia was 865 (3.6%).

### Outcome

The main outcome was height gain (cm) during childhood, defined as the growth in height from birth to five years of age. Height gain was estimated by subtracting height at birth from the height at 5 years of age. We also calculated Z-scores of height and weight at birth, 18 months, 3, 4 and 5 years using population means and standard deviations in each age group according to Swedish standardized growth curves.[15, 16]

### Covariates

Maternal parity, age, body mass index (BMI) in early pregnancy, height, smoking habits in early pregnancy, mothers’ country of birth and level of education, maternal diabetes and infant’s sex, paternal smoking four weeks after birth of the child, exclusive or partial breastfeeding at 6 months and childhood obesity at 5 years were used as covariates. Maternal diabetes was defined as pre-gestational or gestational diabetes and identified through ICD-10 codes (O24.0, O24.1, O24.3, O24.4, O24.9, E10-11 and E13-14). Pre-gestational diabetes was also defined by a check-box in the Birth Register. Childhood obesity was defined as BMI ≥19.3 in boys and ≥19.2 in girls, which is more than 2 standard deviations (SD) over population average according to Swedish reference for 5 year old children.[17]

### Statistical analyses

Demographic and clinical variables were compared between pregnancies exposed to severe and mild preeclampsia and unexposed by one-way ANOVA with Tukey post hoc test for continuous covariates and qui square test for categorized covariates.

Children’s pattern of height gain was compared longitudinally with Z-scores. Height measurements at birth, 18 months, 3, 4 and 5 years were standardized to population growth curves in boys and girls separately. [15, 16] Population mean in each age group was subtracted from the observed value, which was then divided by the population standard deviation. The Z-score at birth was further standardized by gestational age in weeks. Mean Z-scores of height with 95% CI were calculated at each time point in children exposed to mild and severe preeclampsia and unexposed to create line graph. Because of the partly missing data at 18 months, 3 and 4 years of age in children born 2000–2003 (see Table 1) we re-analyzed the z scores in a population of children born 2003–2007 but this did not change the results.

Height gain (cm) during the first five years of life was calculated and the difference in mean height gain between exposed and unexposed was estimated with linear regression. Adjustments were made in three steps. Step one (model 1) included parity, maternal age, early pregnancy BMI, height, maternal smoking habit in early pregnancy, mothers’ country of birth, level of education, diabetes, and infant’s sex, breastfeeding at 6 months, paternal smoking habit four week after birth and childhood obesity. In the attempt to estimate if the effect of preeclampsia was mediated through decreased birth weight, step two and three were introduced. In step two (model 2) the standardized birth weight for gestational age was added into the model categorized to small, appropriate, and large for gestational age infants, defined by the 10^th^ and 90^th^ percentiles according to the Swedish sex-specific fetal growth curve. [15] Finally, in step three (model 3) the gestational age in days at birth was further added into the model. Maternal BMI and height were used as continuous variables whereas other variables were categorized according to Tables 2 and 3. To minimize the risk of a remaining effect modification by birth weight for gestational age, the regression was repeated in a population restricted to children appropriate for gestational age (AGA), with adjustments to all the covariates in model 1 and gestational age in days.

**Table 2:** Maternal characteristics in pregnancies complicated with severe and mild preeclampsia and women without preeclampsia.

	Severe preeclampsia		Mild preeclampsia		No preeclampsia	
	(N = 179)		(N = 686)		(N = 22,898)	
	N	% or mean (SD)	N	% or mean (SD)	N	% or mean (SD)
Age (years)						
< 25 years	21	^ɵ^ 11.7%	85	^ɵ^ 12.4%	2737	12.0%
25–29 years	57	37.4%	235	34.3%	6954	30.4%
30–34 years	56	31.3%	227	33.1%	8475	37.0%
≥ 35 years	35	19.6%	139	20.3%	4732	20.7%
BMI in early pregnancy						
Mean (kg/m^2^)	151	^a^ 26.0 (4.9)	583	^a^ 26.8 (5.5)	19,501	24.5 (4.3)
Missing	28	15.6%	103	15.0%	3397	14.8%
Height						
Mean (cm)	174	^ɵ^ 166.0 (5.8)	659	^ɵ^ 166.6 (6.3)	22,120	166.7 (6.3)
Missing	5	2.8%	27	3.9%	778	3.4%
Mothers country of birth						
Nordic counties	155	^ɵ^ 86.6%	635	^a^ 92.6%	19,985	87.3%
European other	7	3.9%	14	2.0%	768	3.4%
Asian countries	9	5.0%	23	3.4%	1521	6.6%
Other	8	4.5%	14	2.0%	623	2.7%
Parental smoking ^†^						
Maternal	10	^ɵ^5.6%	51	^ɵ^ 7.4%	1594	7.0%
Missing	9	5.0%	25	3.6%	725	3.2%
Paternal	13	^ɵ^ 8.2%	50	^ɵ^ 7.7%	1793	8.6%
Missing	20	11.2%	40	5.8%	2060	9.0%
Education (years)						
≤9	24	^ɵ^ 13.4%	102	^a^ 14.9%	3594	15.7%
10–14	120	67.0%	480	70.0%	14,351	62.7%
≥15	30	16.8%	86	12.5%	4032	17.6%
Missing	5	2.8%	18	2.6%	921	4.0%
Diabetes ^§^	12	^a^ 6.7%	32	^a^ 4.7%	311	1.4%

P values calculated with one-way ANOVA with Tukey post hoc test and qui square test.

^a^ p < 0.001 compared to unexposed.

^ɵ^ p > 0.05 compared to unexposed.

^†^ Maternal smoking daily in early pregnancy and paternal smoking one month after the birth of the child.

^§^ Pre-gestational or gestational diabetes.

**Table 3:** Characteristics of children exposed to mild and severe preeclampsia and unexposed to preeclampsia.

	Severe preeclampsia		Mild preeclampsia		No preeclampsia	
	(N = 179)		(N = 686)		(N = 22,898)	
	N	% or mean (SD)	N	% or mean (SD)	N	% or mean (SD)
Firstborn	122	^a^ 68.2%	445	^a^ 64.9%	9731	42.5%
Girl	94	^ɵ^ 52.5%	350	^ɵ^ 51.0%	11,093	48.4%
Breastfed at 6 months *	96	^a^ 53.6%	452	^a^ 65.9%	15,730	68.7%
Missing	20	11.2%	35	5.1%	2099	9.2%
Birth weight						
Mean (grams)	179	^a^ 2372 (920)	686	^a^ 3318 (674)	22,890	3567 (561)
SGA †	65	^a^ 36.3%	137	^a^ 20.0%	2228	9.7%
AGA †	98	54.7%	484	70.6%	18,244	79.7%
LGA †	16	^ɵ^ 8.9%	64	^ɵ^ 9.3%	2330	10.2%
Gestational age at birth						
Mean (weeks)	179	^a^ 34.6 (3.6)	686	^a^ 38.5 (2.0)	22,880	39.3 (1.8)
Preterm (< 37 weeks)	113	^a^ 63.1%	83	^a^ 12.1%	1212	5.3%
Height (Z-scores)						
Birth (length)	167	^a^ -0.5 (1.5)	681	^b^ 0.1 (1.4)	22,698	0.2 (1.2)
At 5 years	179	^b^ -0.1 (1.0)	686	^b^ 0.2 (1.1)	22,896	0.1 (1.0)
Height gain (cm) §	167	^a^ 65.1 (5.2)	682	^a^ 62.0 (4.4)	22,785	60.7 (4.3)
Obesity at 5 year ǂ	5	^ɵ^ 2.8%	28	^ɵ^ 4.1%	852	3.7%

P values (mild and severe preeclampsia compared with unexposed) calculated with one-way ANOVA with Tukey post hoc test and qui square test.

^a^ p < 0.001 compared to unexposed.

^b^ p < 0.05 compared to unexposed.

^ɵ^ p > 0.05 compared to unexposed.

* Exclusively or partially breastfed.

^†^ Small for gestational age (SGA) defined by standardized birthweight for gestational age less than 10^th^ percentile, appropriate for gestational age (AGA) 10^th^– 90^th^ percentile and large for gestational age (LGA) more than 90^th^ percentile, according to the Swedish sex-specific fetal growth curve.[15]

^§^ Height gain defined as length at birth subtracted from the height at 5 years of age.

^ǂ^ Defined as BMI ≥19.3 in boys and ≥19.2 in girls at 5 years according to Swedish standard curves.[17]

SPSS software (version 22) was used for the analysis.

The study was approved by the Regional Ethical Review Board in Uppsala (2013–351).

## Results

In pregnancies complicated with preeclampsia (severe and mild), mothers had higher mean BMI than in pregnancies without preeclampsia whereas maternal height was similar in women with and without preeclampsia. (Table 2) Further, mothers with mild preeclampsia were more often born in Nordic countries and had slightly shorter education than those without preeclampsia. Lastly, mothers with severe and mild preeclampsia had more often pre-gestational or gestational diabetes than mothers without preeclampsia.

**Fig 1:**
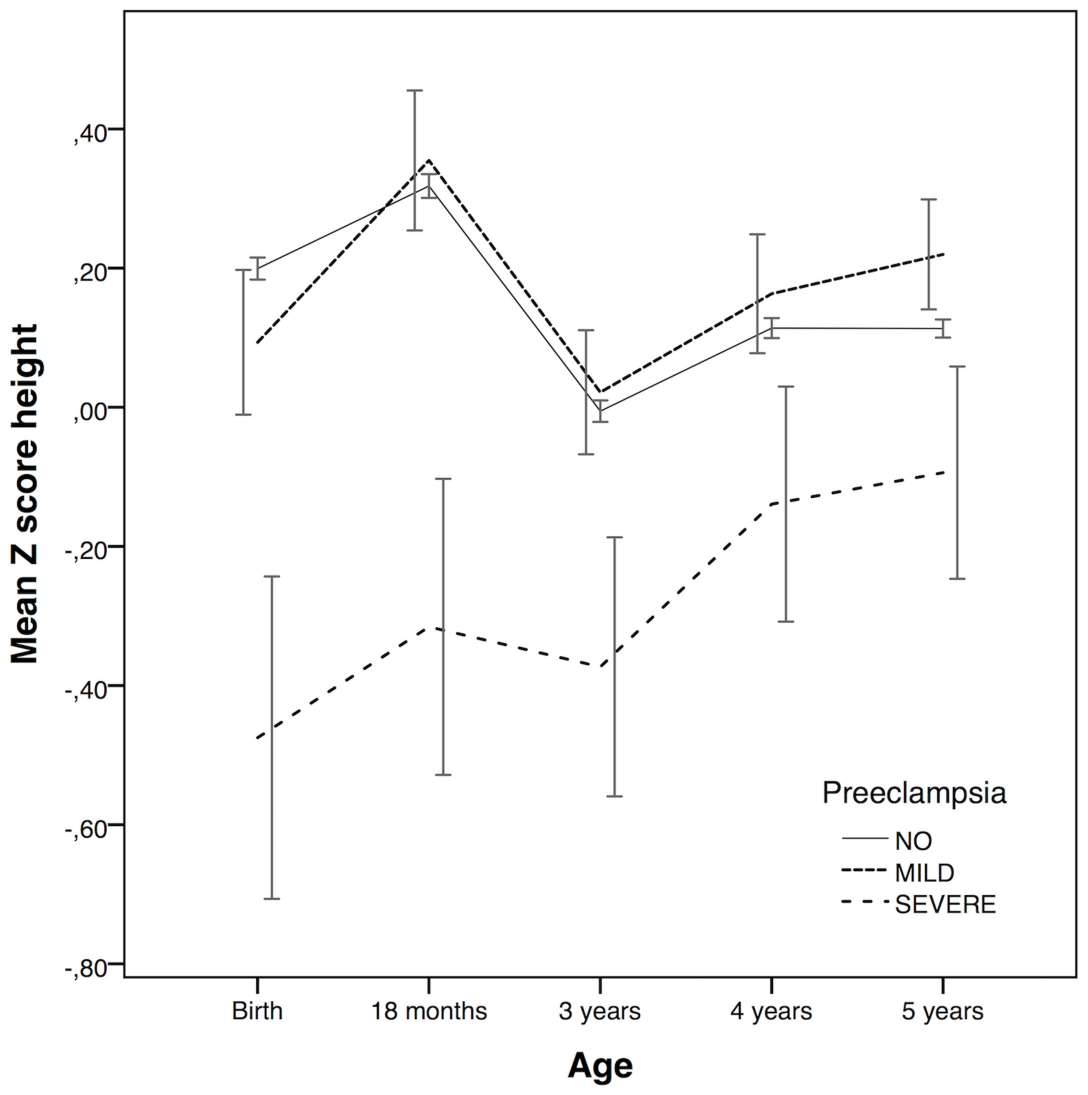
Mean Z scores of height with 95% confidence intervals at different ages in children exposed to mild and severe preeclampsia and unexposed to preeclampsia.

Children exposed to preeclampsia were more often their mother’s first born child and were less often breastfed at the age of six months than unexposed. (Table 3) Further, children exposed to preeclampsia were more often born SGA than unexposed, especially those exposed to severe preeclampsia. Among children exposed to severe preeclampsia, 63.1% were born preterm and they were on average shorter at birth (45.6 cm) than unexposed (50.8 cm) children. Although children exposed to severe preeclampsia grew more than unexposed from birth to 5 years, they were shorter at the age of five than unexposed and with an average height below the population mean. (Fig 1) Among children exposed to mild preeclampsia, only 12.1% were born preterm and they were only slightly shorter at birth than unexposed. However, children exposed to mild preeclampsia were taller than unexposed and above the population mean at the age of five. Obesity at the age of five did not differ between groups.

After adjustments for maternal parity, age, BMI, height, country of birth, smoking, level of education and diabetes, paternal smoking, child’s sex, infant’s breastfeeding and childhood obesity, the difference in mean height gain between exposed and unexposed was slightly attenuated from 1.9 cm (95% CI 1.6–2.1 cm) to 1.6 cm (95% CI 1.3–1.9 cm) (Table 4). This analysis was also repeated with stratification by sex, and the result did not imply an effect modification by sex (not shown in table). With further adjustments for birth weight for gestational age the estimates were similar, but adjustment for gestational age in days substantially decreased the estimates, 0.5 cm (95% CI 0.1–0.7 cm). When the population was restricted to children with birth weight appropriate for gestational age (10^th^– 90^th^ percentile according to the Swedish sex-specific fetal growth curve) [15], the fully adjusted estimate of height gain was 0.5 cm (95% CI 0.1–0.8 cm) larger in exposed than unexposed (adjusted for covariates in model 1 and gestational age in days, result not shown in table).

**Table 4:** The mean difference in height gain between children exposed and unexposed to preeclampsia.

	Mean difference in height gain in cm (95% CI)			
	Crude	Adjusted 1 *	Adjusted 2 †	Adjusted 3 ǂ
Preeclampsia total	1.9 (1.6–2.2)	1.6 (1.3–1.9)	1.5 (1.2–1.9)	0.5 (0.1–0.7)
Mild preeclampsia	1.3 (1.0–1.6)	1.0 (0.6–1.4)	0.9 (0.6–1.3)	0.4 (0.0–0.7)
Severe preeclampsia	4.4 (3.7–5.0)	4.3 (3.6–5.0)	4.2 (3.4–4.9)	1.0 (0.3–1.7)

Height gain defined as length at birth subtracted from the height at 5 years of age.

* Model 1: Adjusted for maternal parity, age, early pregnancy BMI, height, country of birth, smoking in early pregnancy, level of education and diabetes, paternal smoking at 4 weeks after birth, child’s sex, infant’s breastfeeding at 6 months and child’s obesity at 5 years of age.[17]

^†^ Model 2: Adjusted as model 1 with the addition of being born small, average or large for gestational age.[15]

^ǂ^ Model 3: Adjusted as model 2 with the addition of gestational age at birth in days.

## Discussion

In this population-based prospective study we could show that exposure to preeclampsia was associated with accelerated height gain during early childhood. The association seemed stronger in children exposed to severe than mild preeclampsia. The association was not confined to children born SGA, but the difference in early childhood height gain between exposed and unexposed may partly be explained by shorter gestational age at birth.

### Strengths and limitations

The major strength of the study was the large number of included children that made it possible to estimate the association between preeclampsia and childhood height gain. The exposure data was collected before measurement of the outcome which precludes recall bias, and the population-based design makes the results generalizable. Further, we had available information on many possible confounding factors, including maternal parity, height and smoking.[18] Information was also available on several covariates related to the metabolic syndrome, including maternal BMI, maternal diabetes,[19] breastfeeding,[18] and children’s obesity.[20] However, we lacked information on maternal gestational weight gain and the family diet, factors that may have affected the association.[18] Further, we lack information on paternal height that seems to be associated with children´s height gain, especially between the age of two and five years.[21] Residual confounding by paternal covariates such as height or weight cannot be excluded. The height measurements were registered as whole numbers and therefore a bias related to rounding cannot be excluded. When we studied the gain in height from birth to five years in children exposed and unexposed to preeclampsia, we chose to adjust for gestational age at birth. This seemed reasonable since preeclampsia is strongly associated with preterm birth and therefore shortness at birth. However, the severity of preeclampsia disorder may be reflected by low gestational age and therefore some over adjustment may have occurred.

### Comparisons to earlier studies

A pattern of accelerated post-natal growth (height and weight gain) is previously described in children born SGA.[9, 10] The majority of children born SGA catch-up in size with their same age peers within the first two years of life, but those who do not catch-up have increased risk of short adult stature.[9] The final height in people exposed to preeclampsia has in a previous study not shown to differ from unexposed,[22] whereas gestational age at birth is associated with the final height.[23] In two previous studies height gain in children exposed to preeclampsia is described.[24, 25] Catch-up growth is described in a cohort of 135 preterm SGA infants born to mothers with preeclampsia.[24] Our results of increasing Z-scores in children exposed to severe preeclampsia indicates a pattern of catch-up growth, and one third of those children were born SGA and more than half of them were born preterm. Therefore, our results seem in concert with previous findings of catch-up growth in children born SGA.[26] However, our results suggest that the association is not confined to children born SGA but also seen in children born AGA. Further, children exposed to mild preeclampsia were taller than unexposed children at the age of five. This interesting finding is also observed recently by Byberg *et al* [25] but in their study the finding was confined to boys. In our study sex did not seem to modify the mean difference in height gain between exposed and unexposed. This difference in results may be explained by different methods used for comparing growth in height in our study (increased height in cm from birth to five years) and Byberg study (modelling of Z scores).

### Potential explanation of the association

More than 20 years ago Barker hypothesized that an intrauterine environment of restricted nutrition might alter the physiology or metabolism in the fetal tissue to optimize the growth of key organs.[27] Further, this adaptation could become unfavorable in a high nutritional post-natal environment.[20, 28] Our results indicate that not only children born growth restricted but also those exposed to preeclampsia, may have different physiology or metabolism compared with unexposed children. A common imbalance in growth factor signaling may explain the shown association between prenatal exposure to preeclampsia and childhood height gain.[29, 30] Insulin-like growth factors (IGFs) are found to correlate with both intrauterine [30] and post-natal growth [29], but the evidence that prenatal preeclampsia induces epigenetic changes in IGFs is limited to small case-control studies at present time.[31, 32]

Alternatively, the shown association may be related to a maternal inheritable trait (genetic or environmental) that predisposes the mother to preeclampsia and the child to an accelerated growth pattern. The metabolic syndrome may be a predisposing trait that confounds the association between prenatal preeclampsia and childhood height gain.[20, 33, 34] Adjustments for factors associated with the metabolic syndrome as well as childhood obesity only had a minor impact on the association in our study. Therefore we find it unlikely that confounding by the metabolic syndrome explains the accelerated height gain in childhood seen after prenatal exposure to preeclampsia. However, unknown genetic confounding may explain the association, i.e. that the same sets of genes influence preeclampsia development in mothers and growth in height of their children.

### Conclusion

Our data implies that early childhood growth trajectories are associated with prenatal exposure to preeclampsia, particularly to severe disease. The association was was not confined to children born SGA, but was attenuated after adjustment for gestational age at birth. The observation is mostly interesting on the basis of a possible pathophysiological difference between exposed and unexposed, but the clinical relevance of the observation is uncertain.

## References

[1] Barquera S, Pedroza-Tobias A, Medina C, Hernandez-Barrera L, Bibbins-Domingo K, Lozano R, et al Global Overview of the Epidemiology of Atherosclerotic Cardiovascular Disease. Archives of medical research. 2015;46(5):328–38. Epub 2015/07/03. doi: 10.1016/j.arcmed.2015.06.006 . 26135634 10.1016/j.arcmed.2015.06.006

[2] Wadhwa PD, Buss C, Entringer S, Swanson JM. Developmental origins of health and disease: brief history of the approach and current focus on epigenetic mechanisms. Seminars in reproductive medicine. 2009;27(5):358–68. Epub 2009/08/28. doi: 10.1055/s-0029-1237424 . 19711246 10.1055/s-0029-1237424 PMC2862635

[3] Barker DJ, Winter PD, Osmond C, Margetts B, Simmonds SJ. Weight in infancy and death from ischaemic heart disease. Lancet. 1989;2(8663):577–80. Epub 1989/09/09. . 2570282 10.1016/s0140-6736(89)90710-1

[4] Davis EF, Lazdam M, Lewandowski AJ, Worton SA, Kelly B, Kenworthy Y, et al Cardiovascular risk factors in children and young adults born to preeclamptic pregnancies: a systematic review. Pediatrics. 2012;129(6):e1552–61. Epub 2012/05/23. doi: 10.1542/peds.2011-3093 . 22614768 10.1542/peds.2011-3093

[5] Geelhoed JJ, Fraser A, Tilling K, Benfield L, Davey Smith G, Sattar N, et al Preeclampsia and gestational hypertension are associated with childhood blood pressure independently of family adiposity measures: the Avon Longitudinal Study of Parents and Children. Circulation. 2010;122(12):1192–9. Epub 2010/09/09. doi: 10.1161/CIRCULATIONAHA.110.936674 . 20823385 10.1161/CIRCULATIONAHA.110.936674 PMC5321267

[6] Eriksson JG, Forsen TJ, Kajantie E, Osmond C, Barker DJ. Childhood growth and hypertension in later life. Hypertension. 2007;49(6):1415–21. Epub 2007/04/25. doi: 10.1161/HYPERTENSIONAHA.106.085597 . 17452506 10.1161/HYPERTENSIONAHA.106.085597

[7] Halldorsson TI, Gunnarsdottir I, Birgisdottir BE, Gudnason V, Aspelund T, Thorsdottir I. Childhood growth and adult hypertension in a population of high birth weight. Hypertension. 2011;58(1):8–15. Epub 2011/05/18. doi: 10.1161/HYPERTENSIONAHA.111.170985 . 21576624 10.1161/HYPERTENSIONAHA.111.170985

[8] Forsen T, Eriksson JG, Tuomilehto J, Osmond C, Barker DJ. Growth in utero and during childhood among women who develop coronary heart disease: longitudinal study. BMJ. 1999;319(7222):1403–7. Epub 1999/11/27. . 10574856 10.1136/bmj.319.7222.1403 PMC28284

[9] Karlberg J, Albertsson-Wikland K. Growth in full-term small-for-gestational-age infants: from birth to final height. Pediatric research. 1995;38(5):733–9. Epub 1995/11/01. doi: 10.1203/00006450-199511000-00017 . 8552442 10.1203/00006450-199511000-00017

[10] Ong KK, Ahmed ML, Emmett PM, Preece MA, Dunger DB. Association between postnatal catch-up growth and obesity in childhood: prospective cohort study. BMJ. 2000;320(7240):967–71. Epub 2001/02/07. . 10753147 10.1136/bmj.320.7240.967 PMC27335

[11] Meis PJ, Michielutte R, Peters TJ, Wells HB, Sands RE, Coles EC, et al Factors associated with preterm birth in Cardiff, Wales. I. Univariable and multivariable analysis. American journal of obstetrics and gynecology. 1995;173(2):590–6. Epub 1995/08/01. . 7645639 10.1016/0002-9378(95)90287-2

[12] Odegard RA, Vatten LJ, Nilsen ST, Salvesen KA, Austgulen R. Preeclampsia and fetal growth. Obstetrics and gynecology. 2000;96(6):950–5. Epub 2000/11/21. . 11084184

[13] Wallby T, Hjern A. Child health care uptake among low-income and immigrant families in a Swedish county. Acta Paediatr. 2011;100(11):1495–503. Epub 2011/05/04. doi: 10.1111/j.1651-2227.2011.02344.x . 21535134 10.1111/j.1651-2227.2011.02344.x

[14] Ros HS, Cnattingius S, Lipworth L. Comparison of risk factors for preeclampsia and gestational hypertension in a population-based cohort study. American journal of epidemiology. 1998;147(11):1062–70. Epub 1998/06/10. . 9620050 10.1093/oxfordjournals.aje.a009400

[15] Niklasson A, Albertsson-Wikland K. Continuous growth reference from 24th week of gestation to 24 months by gender. BMC pediatrics. 2008;8:8 Epub 2008/03/01. doi: 10.1186/1471-2431-8-8 . 18307822 10.1186/1471-2431-8-8 PMC2294116

[16] Wikland KA, Luo ZC, Niklasson A, Karlberg J. Swedish population-based longitudinal reference values from birth to 18 years of age for height, weight and head circumference. Acta Paediatr. 2002;91(7):739–54. Epub 2002/08/31. . 12200898 10.1080/08035250213216

[17] Karlberg J, Luo ZC, Albertsson-Wikland K. Body mass index reference values (mean and SD) for Swedish children. Acta Paediatr. 2001;90(12):1427–34. Epub 2002/02/21. . 11853342 10.1111/j.1651-2227.2001.tb01609.x

[18] Regnault N, Botton J, Forhan A, Hankard R, Thiebaugeorges O, Hillier TA, et al Determinants of early ponderal and statural growth in full-term infants in the EDEN mother-child cohort study. The American journal of clinical nutrition. 2010;92(3):594–602. Epub 2010/07/02. doi: 10.3945/ajcn.2010.29292 . 20592134 10.3945/ajcn.2010.29292

[19] Duckitt K, Harrington D. Risk factors for pre-eclampsia at antenatal booking: systematic review of controlled studies. BMJ. 2005;330(7491):565 Epub 2005/03/04. doi: 10.1136/bmj.38380.674340.E0 . 15743856 10.1136/bmj.38380.674340.E0 PMC554027

[20] Wells JC. Body composition in infants: evidence for developmental programming and techniques for measurement. Reviews in endocrine & metabolic disorders. 2012;13(2):93–101. Epub 2012/03/16. doi: 10.1007/s11154-012-9213-9 . 22418619 10.1007/s11154-012-9213-9

[21] Botton J, Heude B, Maccario J, Borys JM, Lommez A, Ducimetiere P, et al Parental body size and early weight and height growth velocities in their offspring. Early human development. 2010;86(7):445–50. Epub 2010/06/29. doi: 10.1016/j.earlhumdev.2010.06.001 . 20580499 10.1016/j.earlhumdev.2010.06.001

[22] Ros HS, Lichtenstein P, Ekbom A, Cnattingius S. Tall or short? Twenty years after preeclampsia exposure in utero: comparisons of final height, body mass index, waist-to-hip ratio, and age at menarche among women, exposed and unexposed to preeclampsia during fetal life. Pediatric research. 2001;49(6):763–9. Epub 2001/06/01. doi: 10.1203/00006450-200106000-00008 . 11385135 10.1203/00006450-200106000-00008

[23] Derraik JG, Lundgren M, Cutfield WS, Ahlsson F. Association Between Preterm Birth and Lower Adult Height in Women. American journal of epidemiology. 2017;185(1):48–53. Epub 2016/12/13. doi: 10.1093/aje/kww116 . 27941067 10.1093/aje/kww116

[24] Beukers F, Cranendonk A, de Vries JI, Wolf H, Lafeber HN, Vriesendorp HC, et al Catch-up growth in children born growth restricted to mothers with hypertensive disorders of pregnancy. Archives of disease in childhood. 2013;98(1):30–5. Epub 2012/11/14. doi: 10.1136/archdischild-2012-302510 . 23148313 10.1136/archdischild-2012-302510

[25] Byberg KK, Oymar K, Eide GE, Forman MR, Juliusson PB. Exposure to preeclampsia in utero affects growth from birth to late childhood dependent on child’s sex and severity of exposure: Follow-up of a nested case-control study. PloS one. 2017;12(5):e0176627 Epub 2017/05/10. doi: 10.1371/journal.pone.0176627 . 28486480 10.1371/journal.pone.0176627 PMC5423584

[26] Bocca-Tjeertes IF, Reijneveld SA, Kerstjens JM, de Winter AF, Bos AF. Growth in small-for-gestational-age preterm-born children from 0 to 4 years: the role of both prematurity and SGA status. Neonatology. 2013;103(4):293–9. Epub 2013/04/04. doi: 10.1159/000347094 . 23548568 10.1159/000347094

[27] Hales CN, Barker DJ. Type 2 (non-insulin-dependent) diabetes mellitus: the thrifty phenotype hypothesis. Diabetologia. 1992;35(7):595–601. Epub 1992/07/01. . 1644236 10.1007/BF00400248

[28] Roseboom TJ, van der Meulen JH, Ravelli AC, Osmond C, Barker DJ, Bleker OP. Effects of prenatal exposure to the Dutch famine on adult disease in later life: an overview. Molecular and cellular endocrinology. 2001;185(1–2):93–8. Epub 2001/12/12. . 11738798 10.1016/s0303-7207(01)00721-3

[29] Baron J, Savendahl L, De Luca F, Dauber A, Phillip M, Wit JM, et al Short and tall stature: a new paradigm emerges. Nature reviews Endocrinology. 2015;11(12):735–46. Epub 2015/10/07. doi: 10.1038/nrendo.2015.165 . 26437621 10.1038/nrendo.2015.165 PMC5002943

[30] Conti E, Zezza L, Ralli E, Caserta D, Musumeci MB, Moscarini M, et al Growth factors in preeclampsia: a vascular disease model. A failed vasodilation and angiogenic challenge from pregnancy onwards? Cytokine & growth factor reviews. 2013;24(5):411–25. Epub 2013/06/27. doi: 10.1016/j.cytogfr.2013.05.008 . 23800655 10.1016/j.cytogfr.2013.05.008

[31] He J, Zhang A, Fang M, Fang R, Ge J, Jiang Y, et al Methylation levels at IGF2 and GNAS DMRs in infants born to preeclamptic pregnancies. BMC Genomics. 2013;14:472 Epub 2013/07/13. doi: 10.1186/1471-2164-14-472 . 23844573 10.1186/1471-2164-14-472 PMC3723441

[32] Anderson CM, Ralph JL, Wright ML, Linggi B, Ohm JE. DNA methylation as a biomarker for preeclampsia. Biological research for nursing. 2014;16(4):409–20. Epub 2013/10/30. doi: 10.1177/1099800413508645 . 24165327 10.1177/1099800413508645

[33] Scioscia M, Karumanchi SA, Goldman-Wohl D, Robillard PY. Endothelial dysfunction and metabolic syndrome in preeclampsia: an alternative viewpoint. Journal of reproductive immunology. 2015;108:42–7. Epub 2015/03/15. doi: 10.1016/j.jri.2015.01.009 . 25766966 10.1016/j.jri.2015.01.009

[34] Li L, Yin J, Cheng H, Wang Y, Gao S, Li M, et al Identification of genetic and environmental factors predicting metabolically healthy obesity in children: Data from the BCAMS study. The Journal of clinical endocrinology and metabolism. 2016:jc20153760. Epub 2016/02/26. doi: 10.1210/jc.2015-3760 . 26913634 10.1210/jc.2015-3760

